# Depression in the Mirror: Depression Severity and Its Link to Negative Judgments of Symptoms

**DOI:** 10.3389/fpsyt.2021.621282

**Published:** 2021-07-23

**Authors:** Federica Visco-Comandini, Andrea Gragnani, Mauro Giacomantonio, Giuseppe Romano, Manuel Petrucci, Francesco Mancini

**Affiliations:** ^1^Associazione Scuola di Psicoterapia Cognitiva (SPC), Rome, Italy; ^2^Department of Human Sciences, Marconi University, Rome, Italy; ^3^Social and Development Psychology Department, Sapienza University of Rome, Rome, Italy

**Keywords:** depression, meta-emotional problem, dysfunctional beliefs, depressive symptoms, non-clinical population

## Abstract

**Background and Objectives:** Depressive states represent a normal and physiological response to the experience of loss. However, it is possible to identify some elements that allow distinguishing physiological depressive states from pathological ones. Over the years, research has confirmed that a stable tendency to negative self-evaluation is a transdiagnostic factor that triggers and amplifies dysfunctional emotional reactivity, thus contributing to the shift from normal to pathological reaction. In this sense, the secondary problem, or *meta-emotional problem*, referring to the negative evaluation of one's depressive state and the consequent dysfunctional attempts to solve it, seems to play an important role. The aim of the present study is to investigate how dysfunctional beliefs and the evaluations of depressive symptoms (*meta-emotional problems*) are related to depression severity.

**Methods:** We asked to a community sample to focus on the depressive symptoms they regard as most distressful and evaluate them through specific questionnaires. One-hundred and eighty nine participants were asked to complete a set of questionnaires: (1) the Meta-Emotional Problem Questionnaire; (2) the Center for Epidemiologic Studies Depression Scale; (3) the Beck Depression Inventory; (4) the Dysfunctional Attitude Scale-24 in order to investigate the relation between dysfunctional beliefs, meta-emotional problems, and depressive symptoms severity.

**Results:** Our results show that higher levels of depression are associated both to more pervasive dysfunctional attitudes and increased evaluation of *meta-emotional problem*. In addition, we conduct a regression analysis to disentangle the impact of the two different measures of depressive symptoms (i.e., BDI-II and CES-D) with two explanatory variables (dysfunctional attitudes and *meta-emotional problem*). Results show that *meta-emotional problem* remains a significant and robust predictor of the severity of depressive symptomatology, while dysfunctional beliefs has a rather weak and non-significant relation with the criterion. In other words, *meta-emotional problem* consistently explains the higher variance of depressive symptoms than dysfunctional beliefs. In conclusion, our study shows a clear link between *meta-emotional problem* and depression severity. This is relevant for clinical practice, as it highlights the importance of specifically targeting beliefs about the depressive condition in cognitive-behavioral treatment of depression, since they represent crucial factors maintaining depressive symptomatologies.

## Introduction

One of the core features of cognitive therapy's perspective on psychopathology is the role of beliefs in engendering and maintaining mental disorders. The pioneers of this approach emphasized how emotional suffering is elicited by specific appraisals of life events (1), and how adverse events that occur early in life can lead to the development of negative self-referential beliefs that generate vulnerability to future psychopathology (2). Over the years research has confirmed that a stable tendency to negative self-evaluation (e.g., frustration and anger toward the self when facing setbacks and failures) is a transdiagnostic factor that triggers and amplifies dysfunctional emotional reactivity (3–6).

Beliefs about emotions also play an important role in psychopathology, as they lead to further emotional experiences that add up to the original ones [“emotions about emotions” (7, 8)]. Ellis (9, 10) highlighted how patients can “disturb themselves about their disturbances,” that is, they suffer for their symptoms, as well as for the negative judgments they hold of their symptoms. This phenomenon has been defined as *secondary problem* or *meta-emotional problem* (9, 10). A well-known example of meta-emotional problem and its involvement in psychopathology has been described by Clark (11) who proposed that panic attacks result from the catastrophic misinterpretation (e.g., “I'm about to die,” “I'm going crazy”) of certain bodily sensations, mainly those involved in normal anxiety responses. This produces a further increase in anxiety and related body sensations, culminating in a panic attack.

As Clark and Beck (12) clearly pointed out (p. 53): “*the greatest differences between clinical and non-clinical anxiety are evident in the secondary, strategic controlled processes responsible for the persistence of anxiety. For the clinical individual further elaboration results in a persistence and even escalation of anxiety, whereas the same processes result in a reduction and possible termination of the anxiety program for the nonclinical person*.” It has been shown that reducing the negative assessment of specific negative emotions related to phobic stimuli (i.e., *meta-emotional problem*) reduces the experience of the aversive emotion itself [i.e., primary problem (13)]. Participants whose *meta-emotional problem* was addressed during therapy also presents a decrease in autonomic arousal (as observed by decreased heart rate and increased heart rate variability) during a second exposure to phobic stimuli (13).

The role of the *meta-emotional problem* in affective disorders can be inferred by research on depressive rumination, considered as a key risk factor and characteristic of clinical depression. For example, Response Styles Theory (RST) (14) views rumination as a trait-like tendency to respond to negative mood through repetitive self-focused thinking. As regards the content of rumination, Nolen-Hoeksema [(14), p. 569] claims that it involves “*repetitively focusing on the fact that one is depressed; on one's symptoms of depression; and on the causes, meanings and consequences of depressive symptoms*.” Conway et al. (15) also emphasize the rumination focus on current depressive distress, especially sadness-related feelings, whereas Teasdale (16) drew a distinction between analytical self-focus (i.e., thinking “about” oneself and one's symptoms) and experiential self-focus (i.e., attending to the direct experience of thoughts, emotions, and sensations) in depression, highlighting the detrimental effects of the former, and the beneficial ones of the latter in maintaining depressed mood. In line with this view, Mindfulness-Based Cognitive Therapy (MBCT), which was specifically designed to promote experiential self-focus in the present moment, has proved effective in the reduction of depressive symptoms and of relapse risk (17–19). Through MBCT, patients in remission from recurrent major depression learn to become more aware of, and to relate differently to, their thoughts, feelings, and bodily sensations, and to change processing strategies used when mood begins to deteriorate. Rumination-focused cognitive-behavioral therapy also leads to improved residual symptoms and remission rates in persistent depression (20, 21).

**Figure 1:**
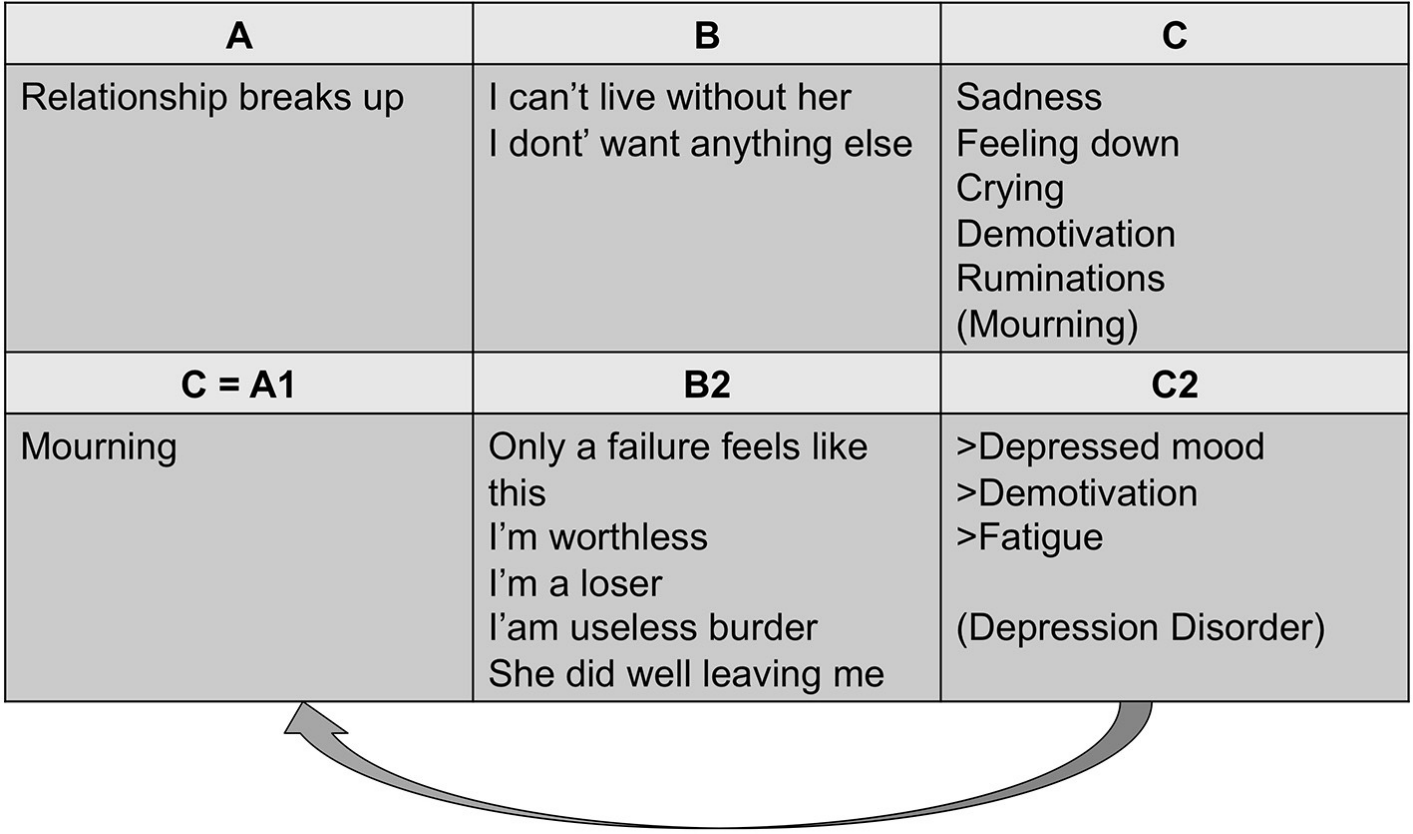
Meta-emotional problem in depression (23).

According to Rainone and Mancini (22), when the physiological correlates of mourning, such as crying and depressed mood, are self-criticized and negatively judged, the “natural” process of loss acceptance is disrupted, leading to the onset of depressive disorder [see also (23); see Figure 1 for a graphical representation of the cognitive process]. The resulting feelings of guilt and shame, considered as the main expression of the *meta-emotional problem* in depressed patients, contribute to a great extent to maintaining pathogenic beliefs, exacerbating the sense of unworthiness, inadequacy and defectiveness, and creating the perception of oneself as being an unbearable burden for other people. This mechanism exacerbates the negative evaluation of the Self (24, 25), thus frustrating fundamental goals such as personal value and lovability, ultimately generating a sense of desperation. Therefore, there is mutual reinforcement between dysfunctional core beliefs that characterized depression [e.g., about the self, the world and the future (26)] and evaluations concerning depressive symptoms (*meta-emotional problem*). While the role of dysfunctional beliefs and attitudes in depressive onset and relapse has been widely emphasized (26–30), the role of the *meta-emotional problem* has not been extensively investigated. Furthermore, it is not clear whether some symptoms are considered as more distressful than others among the variety of depressive expressions. In its early formulation, Beck's cognitive model of depression proposed that certain schemas or cognitive distortions are latent but can be activated by life events that match these schemas, resulting in automatic negative thoughts and depressive symptomatology (26). Later, it was specified that these schemas could be activated by any event (30), or an event can be reactivated by sad mood itself (31, 32). This interpretation highlights the role of specific negative emotions as trigger factors to the development of negative thoughts and depressive symptoms.

To sum up, negative evaluative judgments of symptoms (i.e., meta-emotional problem) and/or about the self have been attributed a prominent role in the genesis and maintenance of depression (26, 33). These judgments have been documented especially within research on depressive rumination, and rumination-focused approaches to the treatment of depression have been developed and tested (17, 21). However, the targeted judgments reflect the transient content of wider ruminative processes that also involve memories, explanations, current problems and their possible solutions, expectations. It is not clear the specific contribution of negative symptom evaluations to negative views of the self and to depression severity, and whether specific symptoms are object of stable negative beliefs.

To the best of our knowledge, no study to date has investigated the relation between negative evaluations (*meta-emotional problem*) of a specific depressive symptom (primary emotional problem) and the severity of depressive-like symptoms.

For these reasons, the aim of the present study is to investigate how dysfunctional beliefs and *meta-emotional problems* are related to depression severity. The study asks to a community sample to focus on the symptoms they regarded as most distressful and evaluate them through a specific questionnaire.

Consistently with the idea that meta-emotional evaluations are central in maintaining sadness and depressive-like symptoms, we anticipate that intensity of depression-like symptoms are better predicted by meta-emotional evaluations rather than by dysfunctional attitudes. In addition, we advance that the association between dysfunctional attitudes and depression intensity is mediated by meta-emotional evaluations. This hypothesis builds on the reasoning that whereas dysfunctional attitudes can certainly promote negative emotions, which ultimately becomes particularly intense and potentially pathological because they are magnified and maintained over time by negative meta-emotional evaluations.

## Materials and Methods

### Participants

One hundred eighty-nine participants [95 female, 94 men; mean age (34.8 ± 12.3) years] were recruited online using Amazon's Mechanical Turk. 2 participants declared to have completed elementary school, 37 middle schools, 154 completed college education, and 2 higher level of education (e.g., PhD). The platform guarantees respondent's privacy and confidentiality. All subjects were Caucasian and native English speakers. Inclusion criteria: (1) aged 18 or older, (2) American citizens. Exclusion criteria: (1) major psychiatric or cognitive problems requiring immediate treatment, (2) organic illness, and (3) substance abuse.

### Questionnaires

After preliminary questions related to socio-demographic information, the following questionnaires were administered:

1. The Meta-Emotional Problem Questionnaire (MEPQ). We developed this brief questionnaire *ad hoc* for the purposes of the present study. This measure is meant to tap into judgment about one's own emotional states and attributes. MEPQ has been implemented borrowing items from different validated questionnaires: Beliefs About Emotions Questionnaire (BAEQ) (34), Pathogenic Beliefs Scale (PBS) (35), Beliefs about emotions Scale (BES) (36). It is important to note that participants were instructed to evaluate how much each items describe how they feel about a specific feeling or bodily state associated to sadness or depression. The final version of the questionnaire includes 22 items with which participants respond on a 7-point scale (i.e., 7-totally agree, 1-totally disagree; see Table 1) with a very good internal consistency (α = 0.97). This measure has been used as predictor variable to investigate the role of the *meta-emotional problem*.
2. Center for Epidemiologic Studies Depression Scale (CES-D) (37). It is a widely used self-report scale designed to measure depression in the general population. The test comprises 20 items, and provides cut-off scores that identify individuals at risk for clinical depression, with good sensitivity specificity and high internal consistency (α = 0.89) in the present sample, Lewinsohn et al. (38). This measure has been used as a criterion to investigate the relation between severity of depressive symptom, meta-emotional problem, and dysfunctional beliefs.
3. Beck Depression Inventory (BDI-II) (39). It is a well-known multiple-choice self-report inventory designed to measure the severity of depression in adults and adolescents. It includes 21 items measuring somatic, cognitive, and behavioral aspects of depression in the last two weeks, as operationalized in the DSM-IV (40). Each item is scored on a four-point scale (α = 0.95).
4. Dysfunctional Attitude Scale-24 (DAS-24) (41, 42). The DAS-24 assesses dysfunctional beliefs expected to reflect a person's self-evaluation. Dysfunctional beliefs have repeatedly been shown in predicting the onset, recurrence and severity of depression (43– 46). The DAS-24 has 24 statements were participants respond on a 7-point scale (i.e., 7-totally agree, 1-totally disagree, α = 0.90). This measure has been used as a criterion to investigate the relation between severity of depressive symptom and *meta-emotional problem* and dysfunctional beliefs.

**Table 1:** Means, standard deviations, and reliability.

	**M**	**SD**	**α**
BDI_II	17.93	13.56	0.95
CES-D	42.47	11.32	0.89
MPEQ	82.74	28.83	0.97
DAS	98.40	20.76	0.90

Descriptive statistics of the measures described above are reported in Table 2.

**Table 2:** Meta-emotional problem questionnaire (MEPQ).

	**List of items**	**MEAN (SD)**
1.	I am a total failure	3.58 (1.75)
2.	I am fundamentally unlovable	3.52 (1.75)
3.	I am worthless	3.50 (1.83)
4.	I am a burden to others	3.90 (1.79)
5.	I am stupid person	3.18 (1.70)
6.	I am going against my moral principles	3.14 (1.73)
7.	Others will hurt, abuse, humiliate, cheat, or manipulate me	3.65 (1.67)
8.	I am different from other people, isolated from the rest of the world, and/or not part of any group or community	4.29 (1.74)
9.	I do not have the right to feel bad when others who have more serious problems than mine	3.99 (1.59)
10.	I am vulnerable, hopelessness	3.92 (1.69)
11.	I am unable to handle everyday responsibilities in a competent manner without considerable help from others	3.68 (1.70)
12.	I do not deserve to be cared for and feel protected	3.49 (1.77)
13.	Others will refuse me	3.96 (1.71)
14.	When I am sick, I cannot help others	4.31 (1.57)
15.	I am a bad person	3.22 (1.66)
16.	I am an inferior person	3.80 (1.73)
17.	I am a failure because I could not make parents or significant others happy	3.77 (1.79)
18.	I am incompetent	3.51 (1.67)
19.	I am inefficient	3.90 (1.74)
20.	Others will judge me negatively	4.23 (1.72)
21.	I am morally responsible for my state	4.24 (1.75)
22.	I'm putting my close relationships at risk	3.98 (1.76)

### Procedure

Participants were asked to complete an introductory assessment on their psychological state (psychotherapy, pharmacotherapy) either in the past or in the present. Subsequently, participants were asked to indicate one depressive symptom among the list of the symptoms indicate in the DSM-5 which cause suffering (depressed mood; diminished interest or pleasure in activities; significant weight loss/weight gain; insomnia/hypersomnia; psychomotor agitation/retardation; fatigue; feeling of worthlessness/excessive guilt; diminished ability to think or concentrate; recurrent thoughts of death). In addition, participants were asked to specify the intensity of the suffering caused by the selected symptom. Successively, as mentioned above, participants fulfilled the meta-emotional questionnaire considering the selected depressive symptom (“*Feeling [chosen symptom] makes me believe that*.”). This questionnaire was used to investigate the *meta-emotional problem* related to the chosen symptoms. Participants were subsequently asked to complete the BDI-II, CES-D, and DAS-24 questionnaires.

### Data Analysis

The relation between depressive symptoms, dysfunctional beliefs, and the meta-emotional problem was investigated using Pearson's correlations, multiple regression analysis, and mediational analysis. Analyses were conducted using PROCESS macro for SPSS (model 4) (47).

Furthermore, to ascertain that the effect we could find was reliable, we also conducted a sensitivity analysis. It indicated that, with a sample of 189 participants and a statistical power of 0.90, the minimum *f*^2^ had to be equal to 0.06.

## Results

### Percentages of Selected Symptoms

We first looked at the distribution of selected symptoms to evaluate which symptoms were chosen more often. Results showed that *depressed mood* was the most chosen (*N* = 57; 30.2%), followed by *feeling restless* and *fatigue or loss of energy* (both *N* = 30, 15.9%), *insomnia/hypersomnia* (*N* = 23; 12.2%), *feeling worthless or guilty* (*N* = 17, 9.0%), *decrease interest or pleasure* and *feeling slowed down* (both *N* = 9, 4.8%), *diminished ability to think or concentrate* and *recurrent thoughts of deaths* (both *N* = 7, 3.7%).

### Correlations

**Figure 2:**
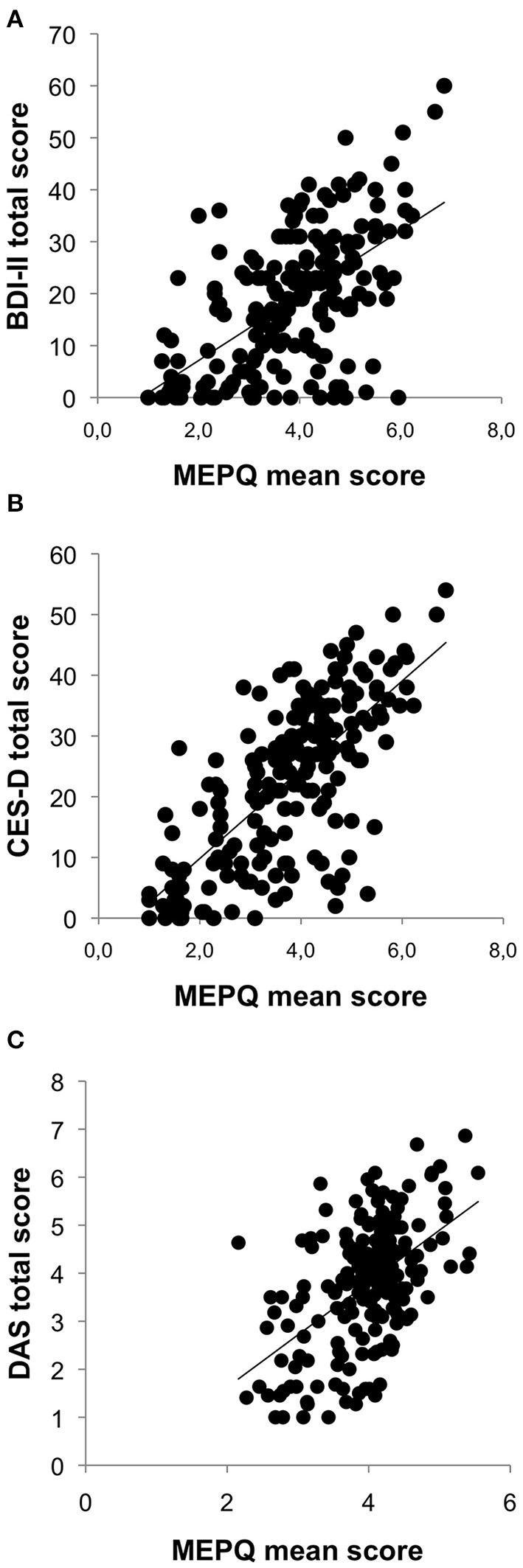
Linear correlation between MEPQ and BDI-II total score **(A)**, MEPQ and CES-D total score **(B)**, and MEPQ and DAS total score **(C)**.

A significant relation between the two measures of in depressive symptoms and the intensity of *meta-emotional problem* emerged [BDI-II: *r* = 0.618, *p* < 0.001 (Figure 2A); CES-D: *r* = 0.676, *p* < 0.001 (Figure 2B)]. The relation between dysfunctional beliefs and the intensity of *meta-emotional problem* (DAS: *r* = 0.526, *p* < 0.001) and between the intensity of depressive symptoms and dysfunctional beliefs [BDI_II: *r* = 0.398, *p* < 0.001; CES-D: *r* = 397, *p* < 0.001 (Figure 2C)] were also statistically significant. Therefore, both depression severity and dysfunctional self-evaluations are clearly linked to the presence of negative judgments toward symptoms.

### Multiple Regressions and Mediational Analyses

**Figure 3:**
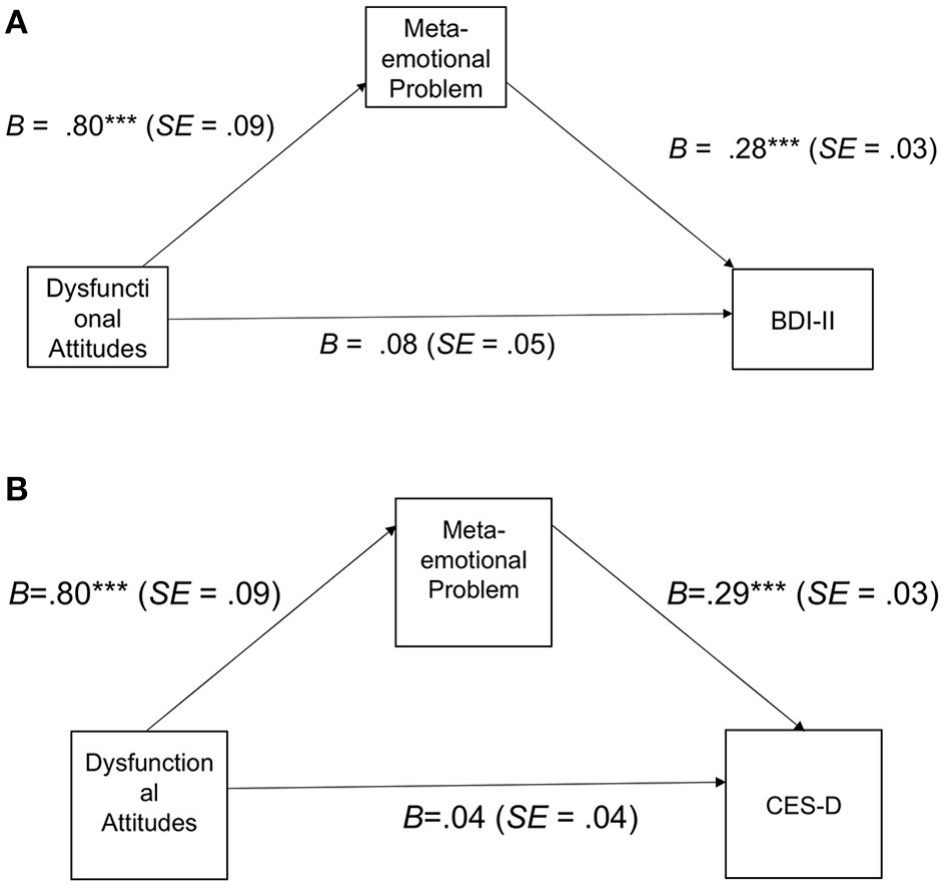
**(A)** Meditational analysis with BDI-II as dependent variable. **(B)** Meditational analysis with CES-D as dependent variable.

To investigate to what extent dysfunctional beliefs and *meta-emotional problem* explain the severity of depressive symptoms, we performed two linear regression analysis (see Table 3). The first regression considers as a dependent variable the BDI-II total score, whereas the DAS total score and *meta-emotional problems* entered as explanatory variables. Results show that, when controlling for *meta-emotional problem*, DAS score is no longer associated with depressive symptoms. In contrast, the association between depressive symptoms and *meta-emotional problem* is significant. We found that the association of *meta-emotional problem* with depressive symptoms remained significant and strong, whereas severity of depressive-like symptoms is not related to DAS scores. These findings thus showed that *meta-emotional problem* is more influential than dysfunctional attitudes in explaining variability of depressive symptoms. The effect sizes of the association between meta-emotional evaluation with BDI-II and CES-D scores were respectively *f*^2^ = 0.37 and *f*^2^ = 0.56. Both values were well above the critical value of 0.06 for the present sample. We can thus consider the parameter of interest to be reliable. We hypothesized that the association between dysfunctional beliefs and depressive symptomatology could be explained by *meta-emotional problem* which could be responsible of magnifying and maintaining the negative emotions originating from dysfunctional beliefs. To test this hypothesis, we conducted two separate mediation models. In the first, we tested whether the association between DAS and BDI-II was mediated by MPEQ. In the second model, we tested whether MPEQ mediated the association between DAS and CES-D. Mediation models are depicted in Figures 3A,B. As expected, the indirect effect of MPEQ between DAS and BDI-II was significant and positive, *B* = 0.22, *SE* = 0.04, 95% *CI* [0.14, 0.31]. Likewise, MPEQ was a significant mediator of the association between DAS and CES-D, *B* = 0.23, *SE* = 0.04, 95% *CI* [0.16, 0.31].

**Table 3:** Hierarchical multiple regression analysis of meta-emotional evaluation and dysfunctional attitudes on depression intensity.

**Criterion**	***Predictors***	***R*^**2**^**	**Adj. *R*^**2**^**	***B* [95% CI]**	**β**	***t***	***p***
BDI-II	MPEQ	0.39	0.38	0.28 [0.21 to 0.34]	0.57	8.38	<0.001
	DAS			0.08 [−0.02 to 0.18]	0.10	1.50	0.14
CES-D	MPEQ	0.46	0.45	0.29 [0.05 to 0.49]	0.64	10.17	<0.001
	DAS			0.04 [0.25 to 0.72]	0.06	0.06	0.37

*BDI-II, Beck Depression Inventory; MPEQ, Meta Questionnaire Problem Questionnaire; DAS, Dysfunctional Attitude Scale-24; CES-D, Center for Epidemiologic Studies Depression Scale*.

From an alternative perspective, it might be argued that the association between MEPQ and depressive symptomatology could be explained by dysfunctional attitudes. In other words, the association between *meta-emotional problem* and depressive symptoms would be explained by the fact that *meta-emotional problem* sustain over time dysfunctional beliefs or could be even considered as specific instance of dysfunctional beliefs. It this would be the case, we should find that DAS mediate the association between MEPQ and depressive symptomatology. To rule out this possibility we conducted further mediational analyses which excluded the mediational role of DAS between MPEQ and BDI-II (*B* = 0.03, *SE* = 0.02, 95% *CI* [−0.01, 0.06] as well as between MPEQ and CES-D (*B* = 0.01, *SE* = 0.02, 95% *CI* [−0.02, 0.05]).

## Discussion

The goal of the present study was to examine whether negative judgments of negative emotions and depressive symptoms (i.e., *meta-emotional problem*) was more strongly associated to depressive symptomatology than dysfunctional attitudes. To this aim, we administered questionnaires measuring the *meta-emotional problem* (i.e., MEPQ), depression severity (i.e., BDI-II, CES-D), and dysfunctional beliefs (i.e., DAS) to a non-clinical sample of adult men and women. Our results show that higher levels of depression are associated with more pervasive dysfunctional attitudes and *meta-emotional problems*. However, regression analysis showed that when entered in the same regression model, only *meta-emotional problem*, explained a significant amount of variance of depressive symptoms measured both with BDI-II and CES-D. More specifically, *meta-emotional problem* remains a significant and robust predictor, while dysfunctional beliefs has a rather weak and non-significant relation with the criterion. This might seem surprising in the light of the prominent role historically attributed to still highly influential dysfunctional beliefs and schemas in the cognitive conceptualization of depression (48, 49), that received conspicuous empirical support in both clinical and neuroscientific fields [for a review, see (50)]. According to the model, depressive vulnerability, in the form of negative beliefs about the self, the others and the future (the negative cognitive triad) is generated by adverse early life events and subsequently triggered by other stressors matching the content of dysfunctional beliefs. Depressive schemas bias perception, attention, and memory in processing of negative information, create recursive mechanisms that reinforce depressed mood (51). The present findings suggest that distorted information processing focused on depressive symptoms plays a prominent role in maintaining and exacerbating depression. Therefore, in addition to rumination and mindfulness based intervention as modulating factors in depression, also *meta-emotional problem* represents a crucial variable for understanding depressive processes.

In the present study, we also advanced and successfully tested the idea that meta-emotional evaluations could mediate the association between dysfunctional beliefs and depressive symptomatology. We speculated, indeed, that dysfunctional beliefs are often related to negative emotion. Such negative emotions can be object of negative *meta-emotional* evaluations thus magnifying and sustaining negative affectivity over time with potentially pathological consequences. Mediational analysis supported this idea and excluded an alternative line of reasoning. That is, we did not find that the relation between MPEQ and depressive symptomatology was mediated by DAS. Thus, it is particularly unlikely that the relation between meta-emotional evaluations and depressive symptomatology could be explained by dysfunctional attitudes hold by an individual.

The present study directly asks the person what is the most disturbing depressive characteristic, detecting specific beliefs that are relevant for the person's goals and wellbeing, rather than general tendencies to negative judgments in the cognitive triad domain. However, it should be noted that our sample is not composed specifically by clinically depressed patients, and the employed design does not allow drawing causal relationships between the investigated factors. Further research is needed to address these issues and extend the present findings.

In conclusion, our study shows a key role meta-emotional problem in the association with depression severity. This is relevant for clinical practice, as it outlines the importance of specifically targeting beliefs about the depressive condition in cognitive-behavioral treatment of depression [e.g., (22, 33)], as they represent crucial factors maintaining depressive symptomatology.

The role of *meta-emotional problem* has been widely emphasized by Rainone and Mancini (22) and Rainone and Mancini (33), who proposed a further integration of the original Beck's cognitive model (48). This revised model highlights new factors contributing to the emergence of depressive symptoms, among which the hypersalient mode, activated by several adverse events, significantly contributes to increase negative appraisals and rumination. According to Rainone and Mancini (33), the latter factors (negative appraisals and rumination) represent significant variables maintaining depressive symptomatology. As it, they represent valuable variables to, respectively, identify depressive schemas and subsequent guide the therapeutic approach. Our results support the hypothesis that the *meta-emotional problem*, together with the dysfunctional beliefs, contributes explaining depressed mood.

Based on these results, we believe that therapeutic approaches treating depression should take into account the role of *meta-emotional problem* in maintaining depressive symptomatology. Therefore, dealing with depressive patients implies using specific therapeutic strategies focusing on *meta-emotional problem* and negative rumination should be encouraged in addition to the standard cognitive-behavioral techniques.

Several limitations need to be acknowledged. Our sample is rather small, therefore further studies are necessary to confirm our results. In addition, regarding the sample type, we also have to highlight the specificity of our sample (all American Caucasian participants) which not permit to generalize our results as a representative group of American citizen. In addition, we did not collect systematic information regarding previous depressive episodes, which could help us highlighting other vulnerability factors. Lastly, the relation between *meta-emotional problem* and severity of depressive symptoms has been investigated through correlation and regression analysis, not taking into account the direction of the relation among variables. Considering the importance of t is clarifying the direction of this relation, further longitudinal studies shall be performed for establishing whether the *meta-emotional problem* represents the cause or the consequence of the depressive disorder.

## Data Availability Statement

The raw data supporting the conclusions of this article will be made available by the authors, without undue reservation.

## Ethics Statement

The studies involving human participants were reviewed and approved by Ethic Committee of Scuola di Psicoterapia Cognitiva (Rome, Italy). The patients/participants provided their written informed consent to participate in this study.

## Author Contributions

FV-C, AG, MP, GR, and FM designed the study. FV-C executed the study. FV-C and MG performed the statistical analysis. FV-C, MP, and MG prepared the manuscript. All authors read and approved the final manuscript.

## Conflict of Interest

The authors declare that the research was conducted in the absence of any commercial or financial relationships that could be construed as a potential conflict of interest.

## Publisher's Note

All claims expressed in this article are solely those of the authors and do not necessarily represent those of their affiliated organizations, or those of the publisher, the editors and the reviewers. Any product that may be evaluated in this article, or claim that may be made by its manufacturer, is not guaranteed or endorsed by the publisher.
